# Pediatric gonadal torsion in radiology: A comprehensive literature and pictorial review using surgically proven cases

**DOI:** 10.1016/j.ejro.2025.100644

**Published:** 2025-03-20

**Authors:** Inácio Freitas, Carolina Soares-Aquino, Pedro Sá, Ana Catarina Silva, Damjana Ključevšek, Sílvia Costa Dias

**Affiliations:** a Department of Radiology, Hospital Dr. Nélio Mendonça, SESARAM, Madeira, Portugal; b Department of Pediatric Surgery, University Hospital Center of São João (CHUSJ), Porto, Portugal; c Department of Radiology, University Hospital Center of São João Porto (CHUSJ), Porto, Portugal; d Department of Radiology, University Children's Hospital Ljubljana, Slovenia; e Department of Medicine, Faculty of Medicine of the University of Porto (FMUP), Porto, Portugal

**Keywords:** Contrastenhanced ultrasonography (CEUS), Testicular torsion, Ovarian torsion, Ultrasonography, Pediatric surgery

## Abstract

Pediatric gonadal torsion is a critical surgical emergency requiring immediate diagnosis and intervention to preserve reproductive capabilities. This review addresses the diagnostic challenges, imaging patterns, and management strategies for both ovarian and testicular torsion, including a brief discussion on the emerging role of Contrast-Enhanced Ultrasound (CEUS), therefore filling a significant gap in the literature. We emphasize the need for a high index of suspicion due to often nonspecific clinical presentations, particularly in ovarian torsion. An accurate and swift diagnosis allows conservative surgical intervention to be offered, which is crucial to maximize gonadal salvage and minimize recurrence. While we highlight CEUS's potential benefits in enhancing diagnostic clarity without ionizing radiation, ultrasound and other modalities such as MRI and CT, have a paramount role in this setting. Future research comparing CEUS with MRI is essential to validate its diagnostic accuracy and effectiveness, potentially revolutionizing acute care diagnostics. Incorporating CEUS into diagnostic workflows, along with a deep understanding of the condition's epidemiology, pathophysiology, and clinical presentation, may probably significantly improve patient outcomes. We detail the characteristic imaging features, diagnostic pitfalls, and differential diagnoses essential for radiologists, with particular relevance for residents and those with limited pediatric radiology exposure. This review aims to bridge existing knowledge gaps and serve as a robust educational tool, facilitating better clinical decision-making and outcomes in pediatric gonadal torsion cases.

## 1 Introduction

Gonadal torsion in the pediatric population is a critical surgical emergency requiring rapid diagnosis and intervention to preserve gonadal function [1], [2]. It includes ovarian torsion in females and testicular torsion in males, each presenting unique diagnostic and treatment challenges [2].

Ovarian torsion is a rare but serious concern, with varied imaging features and nonspecific symptoms often leading to delayed identification and misdiagnosis [2], [3]. A high index of suspicion is essential for prompt treatment to avoid irreversible gonadal damage [3]. Conservative surgical intervention, focusing on detorsion and ovarian salvage, is recommended to maintain future fertility potential [2], [4].

Acute scrotal pain in pediatric patients warrants immediate evaluation due to the high likelihood of testicular torsion [5]. Although the critical window for testicular salvage is traditionally within 6–8 h from symptom onset, emerging evidence suggests that aggressive management can yield favorable outcomes even beyond this timeframe, with survival rates of 90 % in the first 12 h and 54 % between 13 and 24 h from onset [6]. This highlights the need for prompt intervention regardless of pain duration [2], [4], [7], [8]. Managing gonadal torsion, underscores the urgency and complexity of the condition, necessitating rapid diagnostic and surgical responses to optimize patient outcomes [1], [7].

This paper provides a comprehensive pictorial and literature review of pediatric gonadal torsion, discussing both testicular and ovarian torsion simultaneously, thus addressing a gap in existing literature where these entities are typically considered separately.

Our aim is to explore the diagnostic challenges, imaging patterns, and the role of various imaging modalities, including the emerging use of contrast-enhanced ultrasound (CEUS). Additionally, we compare the imaging findings of both conditions to highlight unique features and common pitfalls and review differential diagnoses to serve as a detailed educational resource for radiologists.

## 2 Methods

A retrospective review was conducted of all pediatric cases (patients aged < 18 years) with surgically confirmed gonadal torsion at our tertiary center (CHUSJ) between January 2011 and December 2024. Initially, the surgical records database was queried to identify every case in which gonadal torsion was definitively documented during operative procedures. These cases were then cross-referenced with the CHUSJ Picture archiving and communication system (PACS) to confirm which patients had preoperative images available in digital format. Because CHUSJ is part of the integrated metropolitan pediatric emergency system in Porto, some patients were referred from other hospitals. In those instances, any external imaging transferred with the patient or new imaging obtained at CHUSJ - provided it was stored in the hospital’s PACS - was included for analysis.

For each case, the collected data included: patient age at the time of the episode, sex, presenting symptoms, sequence and pertinent findings of imaging studies (US, CEUS, CT, MRI), preliminary radiological diagnosis, details of the surgical procedure as recorded in the operative notes, the final postoperative diagnosis, patient outcomes, and follow-up information. From these imaging sets, the authors then selected the most illustrative examples that accurately depicted key torsion signs and features. A comprehensive overview of demographic, clinical, and imaging data for all surgically proven cases of ovarian torsion is provided in Supplementary Table 1.

Due to varying clinical scenarios, some cases underwent multiple imaging methods or had recurrent torsion episodes, leading to the same patient potentially appearing more than once in the final set of figures.

Only complete records were included, meaning cases had to show clear documentation of:

− Definitive surgical confirmation of gonadal torsion,
− Preoperative imaging accessible in the PACS, and
− Enough clinical and follow-up information to describe outcomes.

Cases lacking any of these components were excluded from this paper.

This methodology ensured that all included cases represented surgically verified torsion, with corresponding imaging.

Using these data, the authors are also preparing a separate manuscript to evaluate the concordance between preoperative imaging findings and surgical diagnoses at our institution and to compare these results with existing literature.

It is worth noting that all patient identifiers were removed to protect confidentiality.

## 3 Epidemiology

### 3.1 Ovarian

Adnexal torsion encompasses the twisting of the ovary, fallopian tube, or both, around their ligamentous supports, leading to vascular compromise [1], [3], [9]. Ovarian torsion has an incidence of 4.9 per 100,000 in females aged 1–20 years [10], [11], [12], much less frequent than acute appendicitis, which has an incidence of 233 per 100,000 persons [13]. It can affect all age groups, with 15 % of cases occurring in the pediatric population [10], [14], [15], [16], with a bimodal distribution, during the neonatal period and adolescence [14], [15], [17], [18]. Almost 16 % of all cases occur in females under one year of age [10], [15], [18], related to ovarian cysts due to elevated maternal hormone stimulation, acting as secondary lead points [14], [19], [20]. Adnexal torsion accounts for up to 67 % of all torsion cases, with isolated tubal torsion (without ovarian involvement) and isolated ovarian torsion (without tubal involvement) being less common. The incidence of the former is estimated at 1 in 1.5 million females [3], [10], [16], [21].

### 3.2 Testicular

Testicular torsion is the most frequent cause of acute scrotum, accounting for 86 % of cases in adolescents [22]. It involves the twisting of the spermatic cord, impairing blood flow to the testis [22]. The annual incidence is approximately 25 per 100,000 males under 25, with peaks during the perinatal period for extravaginal torsion and during puberty (around 12 years of age) for intravaginal torsion, although it can occur at any age [2], [22], [23].

## 4 Clinical presentation

### 4.1 Ovarian

Diagnosing torsion is challenging due its general symptoms and limitations of physical examinations [9], [10]. The primary symptom is sudden lower abdominal pain, often accompanied by nausea and vomiting due to peritoneal irritation [9], [10], [14]. Other symptoms, such as radiating pain, elevated white blood cell count, fever, and dysuria, can be misleading [9], [14], [15], [24], [25]. Some patients experience gradual onset, with weeks of recurring pelvic pain or intermittent waves of acute pain and nausea, suggesting intermittent torsion [14], [25].

Diagnosing torsion in very young patients is complex as they may not effectively communicate their discomfort [14]. Pelvic examinations are typically reserved for specific cases, such as those with relevant vaginal complaints or who are sexually active, but are not essential for diagnosing torsion [10], [26]. Laboratory tests generally do not provide conclusive evidence [10], although a minor increase in white blood cell count may be observed in some cases [10], [27].

Clinicians frequently rely on US imaging due to the vague clinical presentation [14]. Infants under one year might present with an abdominal mass or feeding intolerance [10], [14], [18], [28]. Occasionally, abnormal prenatal US findings may be the only indication of torsion [14], [28].

### 4.2 Testicles

Testicular torsion typically presents with severe, unilateral testicular pain, often accompanied by nausea and vomiting [7], [22], [29]. Physical examination may reveal the affected testis positioned higher than usual, in a transverse orientation, due to the spermatic cord twisting, and appearing larger due to venous congestion [7], [22]. A key diagnostic indicator is the absence of the cremasteric reflex, which is a clear sign of this condition [7], [22], [30].

## 5 Pathophysiology

### 5.1 Ovarian

Adnexal torsion often results in ovarian ischemia and necrosis, and in rare cases, especially in neonates, it can lead to autoamputation [1], [10], [31]. There's a notable predilection for the right side [9], [32], attributed to the cecum's mobility allowing greater movement of the right ovary compared with the sigmoid colon's fixed position limiting left-sided torsion [9], [14], [21], [24].

Pediatric ovarian torsion is frequently associated with an ovarian mass, typically benign like an ovarian cyst or teratoma, though it can also occur in normal ovaries in up to 25 % of cases [1], [10], [14], [18], [33]. Torsion of normal adnexa is more common in the pediatric population compared to adults, likely due to the longer utero-ovarian ligaments granting excessive mobility [10], [14], [15], [31], [34]. Malignancy in a torsed adnexal mass is extremely rare [10], [14], [33].

The occurrence of torsion follows a bimodal age distribution, aligning with the development of functional ovarian cysts as a result of hormonal stimulation during the first year of life (from maternal hormones during the antenatal period) and at menarche (due to maturation of the hypothalamic-pituitary-gonadal axis) [1], [14], [18], [33]. The timing for surgical intervention varies among individuals, with pain duration beyond 10 h linked to an increased likelihood of tissue necrosis [10].

### 5.2 Testicular

Testicular torsion occurs due to the partial or complete twisting of the spermatic cord, causing increased venous pressure [7], [22]. When this pressure matches arterial pressure, it acutely reduces or halts arterial flow to the testis [7], [22], resulting in loss of testicular function and fertility [7], [22].

**Fig. 1:**
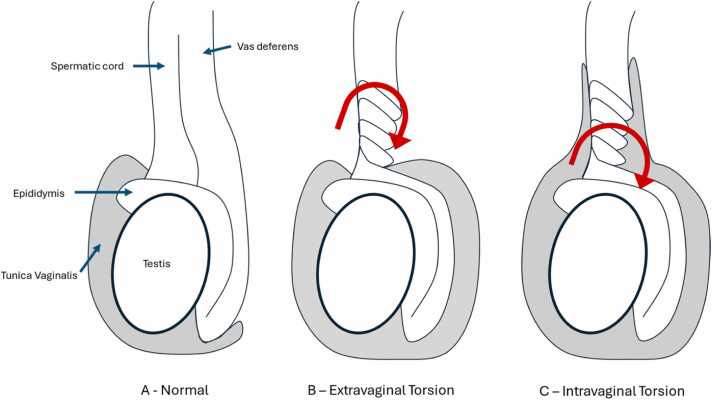
Diagrammatic representation of testicular torsion associated anatomy. Normal anatomy (A). Location of the spermatic cord twisting in extravaginal (B) and intravaginal (C) testicular torsion (shown in gray in the diagram).

Testicular torsion can be either extravaginal or intravaginal (Fig. 1), with the latter being significantly more common [1]. The distinction lies in the location of the twist in the spermatic cord: extravaginal torsion occurs outside the tunica vaginalis, while intravaginal torsion occurs within it [35].

Intravaginal torsion occurs predominantly in adolescents but can also affect neonates [1]. It is attributed to an abnormal attachment of the tunica vaginalis, known as the "bell clapper deformity" [1], [22], [36], which allows the testis and spermatic cord to rotate freely within the tunica vaginalis, making torsion more likely [22].

Extravaginal torsion, can occur during pregnancy (antenatal torsion) or within the first month of life (perinatal torsion) during testicular descent [37]. It results from incomplete fixation of the gubernaculum to the scrotal wall [1], [22], leading to torsion of the testis and tunica vaginalis within the scrotum [1], [35]. About 70 % of these cases occur prenatally and are often difficult to salvage, while the remaining 30 % occur postnatally, with a higher potential for successful intervention [1], [38], [39]. It is estimated that up to 22 % of cases occur bilaterally [39].

## 6 Diagnosis

### 6.1 Ovarian

Diagnosing adnexal torsion is challenging due to its vague and variable clinical presentation and nonspecific imaging findings [10]. It is typically diagnosed through a combination of clinical assessment, patient history, and imaging studies, with laparoscopic exploration being the definitive method for both diagnosing and treating torsion, particularly in the pediatric population [10], [11], [34].

Suprapubic pelvic ultrasonography with color Doppler is the primary imaging tool for diagnosis in pediatric and adolescent patients [10], [14], with an estimated sensitivity and specificity of 84 % and 77 %, respectively [40], [41]. While the absence of vascular flow on Doppler-US strongly suggests torsion, the technique's sensitivity for detecting absent arterial flow is as low as 40–73 % [10], [42]. Transvaginal imaging does not seem to improve diagnostic accuracy [41], [43], and is generally avoided in virginal patients, thus not commonly performed in this group [14], [44].

**Fig. 2:**
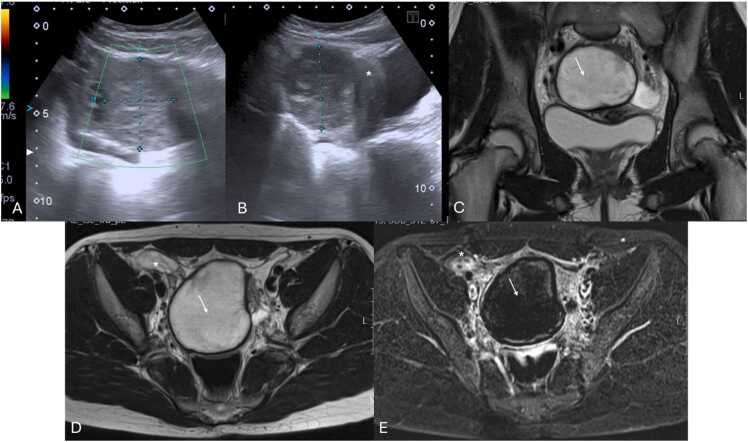
(A and B) Suprapubic Pelvic Ultrasound with Color Doppler performed in the emergency department on a 12-year-old female, revealing an enlarged left ovary (between calipers) located in an abnormal position, adjacent to the uterine fundus (*), with a heterogeneously abnormal echotexture and loss of vascular flow. Given the nonspecific imaging findings, MRI imaging was obtained. Coronal and axial T2-weighted (C and D, respectively) as well as T1-weighted Fat Suppressed (FS) post-contrast subtraction sequence (E) images confirming the presence of an enlarged left ovary (arrows), abnormally positioned in the midline, with loss of follicles and lacking parenchymal enhancement. In (D and E) please note the right ovary with preserved volume, parenchymal follicles and normal enhancement.

Computed Tomography (CT) and Magnetic Resonance Imaging (MRI) are not typically first-line imaging modalities but may be used to clarify anatomy or consider other potential diagnoses when US is inconclusive or unavailable (Fig. 2) [10].

### 6.2 Testicles

**Fig. 3:**
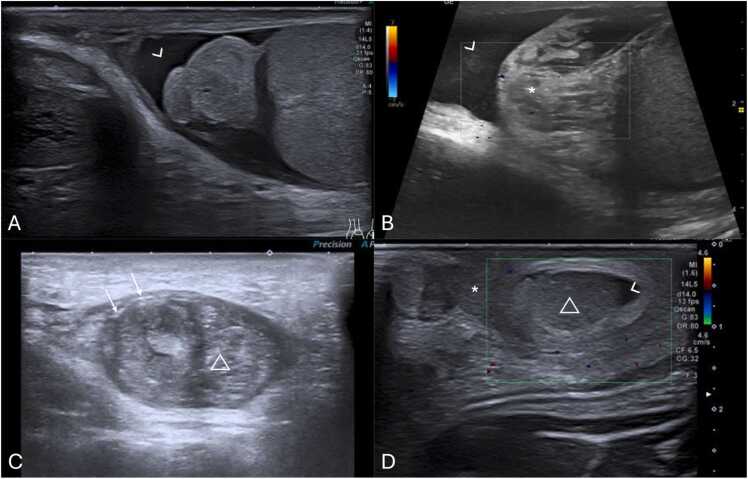
(A–D) Scrotal ultrasound of surgically confirmed testicular torsion in different patients. (A and B) Intravaginal testicular torsion in two different males, depicting a hypoechoic torsed testicle with fluid (arrowhead) surrounding both the testicle and spermatic cord (*), indicating the presence of a bell-clapper deformity. (C and D) Extravaginal testicular torsion in different patients, depicting atrophied testis (△) with an heterogenous parenchymal structure with associated linear calcifications of the tunica albuginea (arrows in C). Notice the presence of fluid extending along part of the subtunica of the torsed testicle (arrowhead in image D), without the presence of fluid surrounding the spermatic cord (* in D), suggestive of extravaginal torsion.

US is highly effective for diagnosing intravaginal testicular torsion (Fig. 3A and B), with a sensitivity of 84–99 % and a specificity of 93–99 %, alongside clinical history and physical examination [22], [45], [46].

The diagnosis of extravaginal testicular torsion (Fig. 3C and D) can often be made through physical examination, with an increase in the consistency of the testicle ("hard" testicle upon palpation) and scrotal hyperemia being key clinical indicators [37]. US is generally used to exclude other rare diagnoses, provided surgical intervention is not delayed [35].

MRI can be used for better characterization in acute scrotal cases where US results are ambiguous and immediate surgical intervention isn't deemed necessary [46]. However, cases suggestive of testicular torsion, marked by uncertain US findings, are typically followed by surgical exploration [46].

## 7 Imaging patterns

### 7.1 Ovarian

#### 7.1.1 Imaging patterns in children and adolescents

In diagnosing ovarian torsion, gray-scale US, Doppler US, and MRI play critical roles in identifying key features [14]. Torsed ovaries can display changes, including internal hemorrhage, stromal edema, infarction, and necrosis, all of which may be visualized on these imaging modalities [14], [47]. Table 1 summarizes the most commonly reported imaging patterns and their approximate frequencies.

**Table 1:** Summarized key imaging findings of ovarian torsion in children and adolescents, and frequency in which they are observed. Modalities that best demonstrate each feature are highlighted.

**Imaging Patterns of Ovarian Torsion in Children and Adolescents with their respective frequency as well as imaging technique in which they are depicted**[3], [41], [50]	
**Imaging Patterns**	**Frequency**
Enlarged ovary on US and/or MRI	**93 %**
Whirlpool sign on Color-Doppler US and/or MRI	**25–91 %**
Accumulation of free fluid in the pouch of Douglas on US and/or MRI	**51–87 %**
Heterogeneous ovary appearance with or without peripheral displacement of follicles by stromal edema and hemorrhage on US and/or MRI	**69–79 %**
Absent ovarian vascularity on Color Doppler-US and/or MRI	**40–73 %**
Simple or complex adnexal mass on US and/or MRI	**56–73 %**
Abnormal ovarian position (midline or opposite pelvis) on US and/or MRI	**34–44 %**
Follicular ring sign on US and/or MRI	**38 %**

**Fig. 4:**
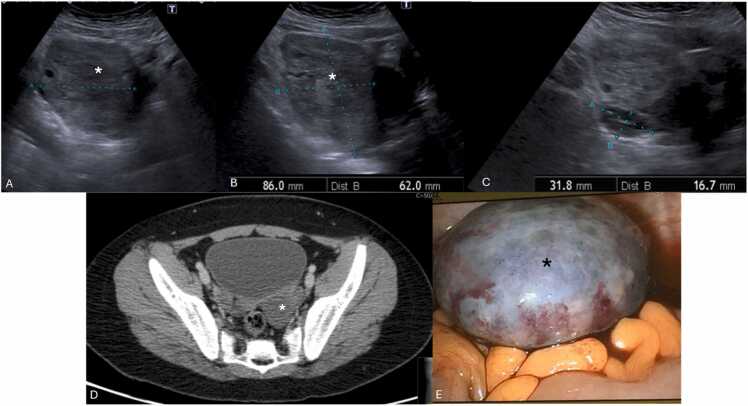
Suprapubic pelvic ultrasound in (A–C) in a 13-year-old female, presenting with sudden pelvic pain, revealed an asymmetrically enlarged left ovary with a volume > 200 cc, with heterogeneous echotexture and loss of normal follicles (* on A and B). Compare with the volume of a normal right ovary (between calipers in C). Due to diagnostic uncertainty, contrast-enhanced pelvic CT (D) was obtained, which shows the enlarged, heterogenous, left ovary in an abnormal position (* in D), with absent central enhancement after contrast administration. Due to clinical and radiological suspicion of ovarian torsion, the patient underwent emergent laparoscopy, and during surgery, a necrotized ovary (* in E) was noted and photographed.

**Fig. 5:**
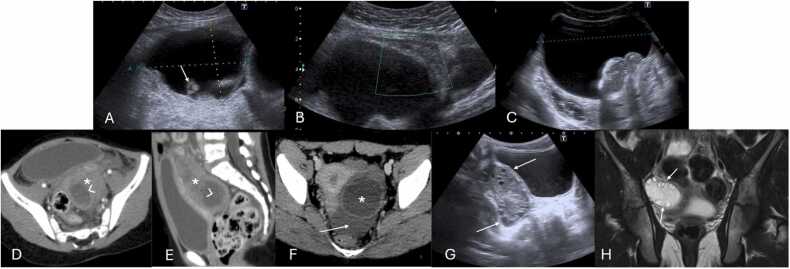
(A and B) 13-year-old female with left pelvic pain, surgically confirmed to be an ovarian torsion. Pelvic ultrasound revealed a large complex cyst (between calipers in A), with multiple thick septations (arrow), and no flow on Color Doppler (B). (C) Large complex mass (between calipers), predominantly cystic, with an internal solid component, in the left ovary, in a 14-year-old female, causing ovarian torsion. This mass was surgically confirmed to be a teratoma. (D and E) Contrast-enhanced pelvic CT of the pelvis of a 12-year-old female depicting an abnormally positioned, enlarged left ovary, containing a large cyst (arrowhead in D and E). Please also note, the loss of the normal enhancement pattern, indicative of torsion (* in D and E). (F) Another contrast enhanced pelvic CT on the axial plane, of a 14-year-old-female showing an enlarged, abnormally positioned, left ovary (*) due to the presence of a large simple cyst (*) and associated free fluid in the pouch of Douglas (arrow). (G and H) Pelvic transabdominal ultrasound (G) and Coronal T2-weighted image of an 11-year-old female with surgically proven right ovarian torsion, depicting heterogeneous ovary appearance with associated peripheral displacement of follicles by stromal edema (arrows).

US findings include unilateral or asymmetric ovarian enlargement (> 20 cc) (Fig. 4) and a heterogeneous ovary appearance due to edema [10], [14]. Other significant indicators seen on US are the presence of a simple or complex adnexal mass (especially rare when larger than 5 cm) (Fig. 5A–F), the accumulation of free fluid in the pouch of Douglas (Fig. 5F), and follicles displaced to the periphery by stromal edema and hemorrhage (Fig. 5G and H) [1], [10], [14]. Abnormal ovarian positioning, in the midline or contralateral hemipelvis, is another reported US finding (Fig. 5) [1], [10], [14].

**Fig. 6:**
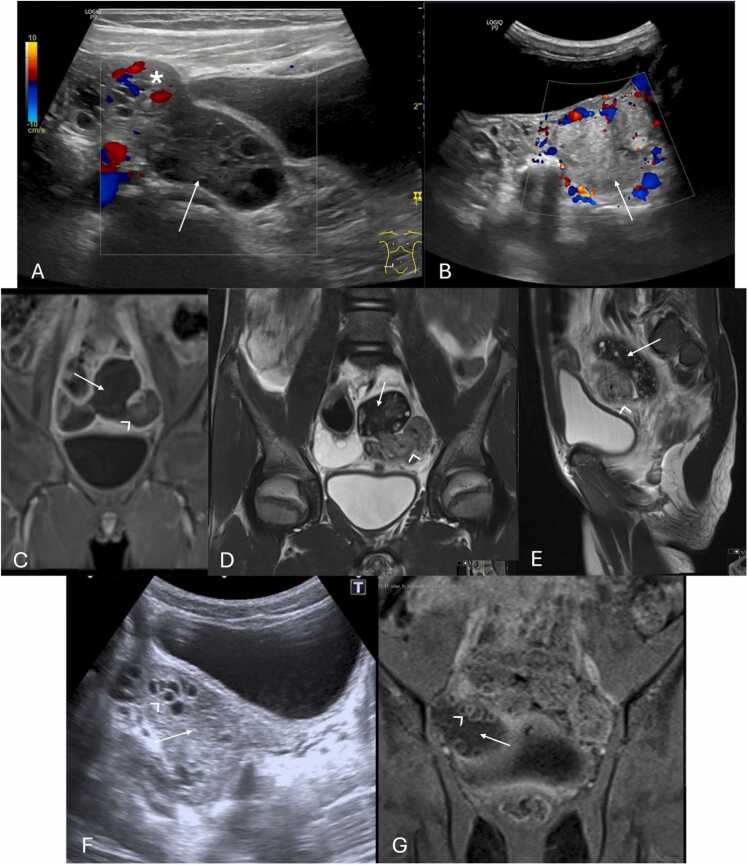
(A and B) Suprapubic pelvic ultrasound with color Doppler in different females, aged 14 and 8 years-old, respectively, depicting different examples of absent ovarian vascularity (arrows), presenting with loss of signal on Color Doppler, highly suggestive of ovarian torsion. In image (A) note the presence of flow within the vascular pedicle (*) of the adjacent fallopian tube, which on Spectral analysis (not shown here) presented with a high-resistive flow (Resistive Index > 0.7), indicating downstream obstruction of the blood flow. To confirm the clinical and radiological suspicion in patient (B), MRI was obtained pre-surgically. Coronal T1-weighted FS post-contrast (C) and T2-weighted (D and E) coronal and sagittal depict an enlarged left ovary (arrows) with loss of normal enhancement pattern after gadolinium injection as well as an abnormal T2 hypointensity. Please note the presence of a twisted ovarian pedicle, representing the whirlpool sign (arrowhead). (F and G) Same patient as in (B–E) 3 years after oophorectomy for surgically confirmed left ovarian torsion, now presenting with pain in the right iliac fossa. Transabdominal pelvic ultrasound and coronal T1-weighted FS post-contrast image depicting an enlarged right ovary (arrow), with heterogeneous parenchyma and peripherally displaced follicles, presenting with a hyperechoic rim, characteristic of the follicular ring sign (arrowhead). It was confirmed during surgery the presence of ovarian torsion.

Color Doppler-US enhances diagnostic specificity by evaluating vascular patterns. It may appear with preserved vascularity, due to the dual vascular supply of the ovaries from both the ovarian and uterine arteries [5], [9], [10]. Alternatively, it may reveal absent ovarian vascularity (Fig. 6A and B), as well as highly specific signs such as the whirlpool sign (twisting of the ovarian pedicle) (Fig. 6C–E) and follicular ring sign (perifollicular hyperechoic rim) (Fig. 6F and G) [1], [10], [14]. These Doppler-specific findings, namely the whirlpool sign and decreased or absent ovarian blood flow, demonstrate pooled sensitivities of 65 % and 53 % and specificities of 91 % and 95 %, respectively [48]. Although traditionally observed on transvaginal US, these findings can also be detected transabdominally [1], [49].

It is worth noting that the effectiveness of the aforementioned findings has not yet been thoroughly assessed in pediatric populations, where transvaginal scans are not typically performed [1], [15], [44].

MRI offers additional diagnostic certainty, particularly in ambiguous cases or when Doppler imaging proves inconclusive [9], [16]. Key MRI findings include ovarian enlargement, stromal edema, internal hemorrhage, and the absence of parenchymal enhancement on contrast-enhanced sequences (Fig. 6 D and E) [9]. Its superior soft-tissue resolution and multiplanar imaging capabilities allow the characterization of adnexal masses, as well as identification of anatomical distortions, such as a twisted pedicle (whirlpool sign) [9], [24].

The presence of an adnexal mass and pelvic fluid, seen on all imaging methods, although frequently associated with ovarian torsion, are associated with a relatively poor diagnostic performance, with pooled sensitivities of 69 % and 55 % as well as specificities of 46 % and 69 %, respectively [48]. Their presence alone may induce false-positive diagnoses and should be interpreted in conjunction with other signs.

**Fig. 7:**
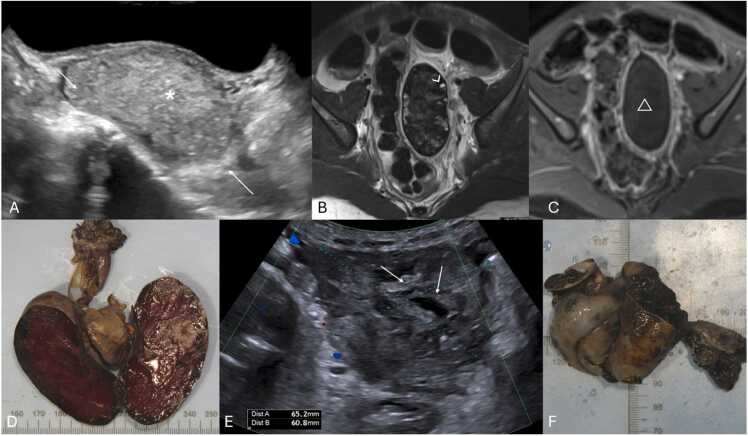
Stromal heterogeneity in different patients indicating hemorrhagic infarction due to advanced ovarian torsion. (A–C) Transabdominal pelvic ultrasound of an 8-year-old female with several days of pain and nausea, depicting an heterogenous ovarian parenchymal echo structure (arrow in A) with scattered areas hyperechogenicity (* in A), indicative of hemorrhagic infarction (which was confirmed at pathologic analysis), associated with loss of normal follicles. MRI of the pelvis was obtained with axial T2-weighted (B) as well as axial T1 FS post-contrast images presenting an abnormally positioned and enlarged left ovary, with a heterogeneous structure and loss of normal parenchymal enhancement (△ in C). Notice the presence of some small follicles in the ovary, that were not seen in US (arrowhead in B). Patient was then submitted to urgent oophorectomy confirming the diagnosis. Pathology specimen of the obtained necrotized left ovary is shown in (D). (E and F) Transabdominal pelvic US accompanied by the respective pathology specimen of a necrotized right ovary in a 13-year-old female presenting with 3 days of severe pelvic pain. US image depicts an enlarged ovary (between calipers in E) with a heterogeneous echo structure due to the presence of scattered areas of increased and decreased echogenicity (arrows in E) consistent with cystic necrotic change, which was confirmed at pathologic analysis (not depicted in F).

Necrotic ovaries may present as more complex or cystic structures due to loss of parenchyma (Fig. 7) [14]. Additionally, acute torsion might cause pelvic tenderness upon probe palpation during US examination [3], [14], [16], with some series reporting a frequency of 78 % [50].

### 7.2 Imaging patterns in fetuses and neonates

**Fig. 8:**
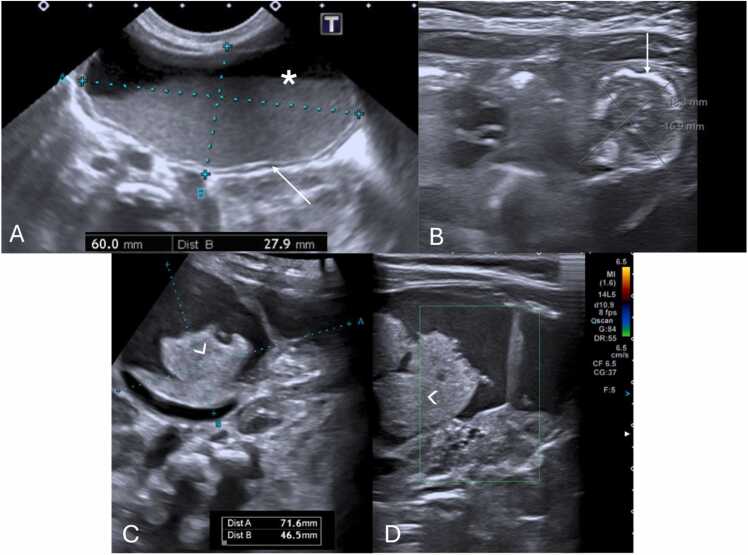
Neonatal ovarian torsion in a 3-day-old infant with a prenatally diagnosed cystic mass with a fluid-debris level. Transverse transabdominal pelvic US image (A) obtained at the 3rd day of life shows a cystic mass (between calipers) with fluid-debris levels (* in A), in the left pelvic region. It was surgically confirmed to be a left ovarian torsion with cystic fluid separated from liquefied hematoma (no pathology images available). Note the presence of an echogenic inner wall and hypoechoic outer wall (arrow in A), typical for the double wall sign, suggesting infarction. (B) Transverse transabdominal pelvic US image of another surgically proven ovarian torsion in a 2-month-old infant who presented with fussiness, shows a rounded soft-tissue mass (between calipers in B) with echogenic peripheral calcifications (arrow in B) seen, suggesting infarction. (C and D) Transabdominal sagittal and transverse pelvic Ultrasound with color Doppler in a 2-week-old infant with a palpable mass in the lower abdomen region, showing a complex cystic (between calipers in C) with thick septa (arrowhead in C and D) without vascular flow and with retractable cloth sign. This mass was described in an obstetric ultrasound as a simple cyst (no previous images available). During exploratory laparoscopy it was confirmed to be a left ovarian torsion.

**Fig. 9:**
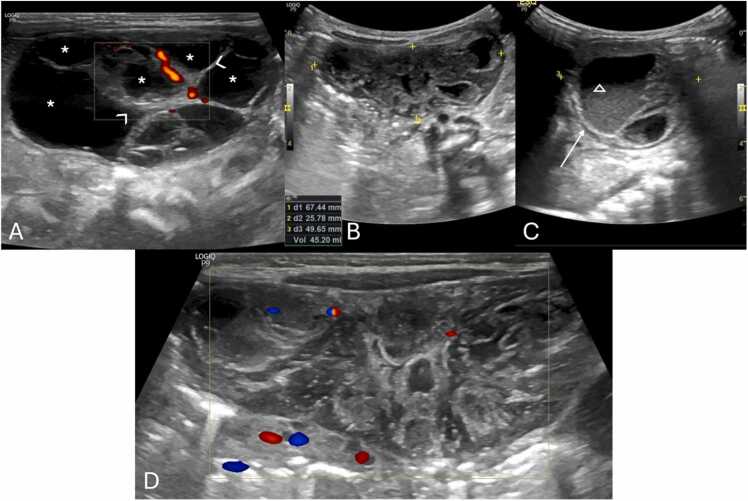
(A–C) Pelvic Ultrasound of a 2-day-old newborn with prenatal diagnosis of pelvic intra-abdominal cystic formations, likely of adnexal origin. The right ovary, in the transverse plane with Power Doppler (A) is show, and is remarkably enlarged, containing multiple central cysts (* in A), separated by septa (arrowheads in A) and preserved vascular flow. Transverse and sagittal (B and C, respectively) pelvic US image depicts the left ovary as a large (volume of 45 cc), complex cystic mass (between calipers in B and C), predominantly solid, containing cystic areas, some with fluid-debris levels (triangle in C). Note the presence of the double wall sign (arrow in C), suggesting infarction. Transverse color-Doppler image (D) of the left ovary shown in (B and C) with general decreased vascular flow. Given the location and imaging findings the diagnosis of left ovarian torsion was suggested, and an exploratory laparoscopy was performed, confirming the diagnosis of left ovarian torsion (no torsion was noted on the right ovary and the cysts were aspirated).

Grayscale US in the context of ovarian torsion in fetuses or neonates may present a more variable appearance compared to older children (Table 2) [14]. Fluid-debris levels within ovarian cysts are a specific sign of ovarian torsion in neonates, indicating separated cyst fluid from a liquefied hematoma (Fig. 8A) [14], [28], [51], [52]. Increased cyst wall echogenicity, representing calcification, also suggests infarction (Fig. 8 A and B) [1], [14], [51]. The evolution of a cyst from simple to complex in sequential USs heightens the suspicion for in-utero torsion, which is typically clinically asymptomatic and detected during routine obstetric US (Fig. 8C and D) [14], [53], [54]. Neonatal torsion may rarely manifest with vomiting, fever, or leukocytosis [14], [28], [55]. US reveals complex cystic masses marked by multi-septation, echogenic debris, and fluid-fluid levels (Fig. 9) [1], [52], [56]. Calcification is occasionally observed, indicating chronicity of the condition (Fig. 9C) [1], [51].

**Table 2:** Summarized imaging findings of ovarian torsion in fetuses and neonates, and frequency in which they are observed.

**Imaging Patterns of Ovarian Torsion in Fetuses and Neonates with their respective frequency**[51], [57]	
**Imaging Patterns**	**Frequency**
Large ovarian cysts (> 5 cm)	**90 %**
Complex cystic masses with multi-septation and echogenic debris	**27–82 %**
Double wall sign	**64 %**
Evolution from simple to complex cysts in sequential USs	**9–57 %**
Fluid-debris / fluid-fluid levels within ovarian cysts	**45 %**
Extrapelvic location of torsed ovaries	**34 %**
Calcification in the cyst wall	**18 %**
Change in the location of the mass	**11–18 %**

Postnatal radiographs can show a round soft-tissue opacity in the abdomen or pelvis, sometimes with a thin peripheral calcification layer [14], [51]. A change in the location of this mass on subsequent exams should raise suspicion for ovarian torsion [14].

Diagnostic pitfalls of neonatal torsion include the extrapelvic location of torsed ovaries, possibly leading to misdiagnoses such as appendicitis or abscess [1], [51], [52].

### 7.3 Testicular

#### 7.3.1 Extravaginal torsion

**Fig. 10:**
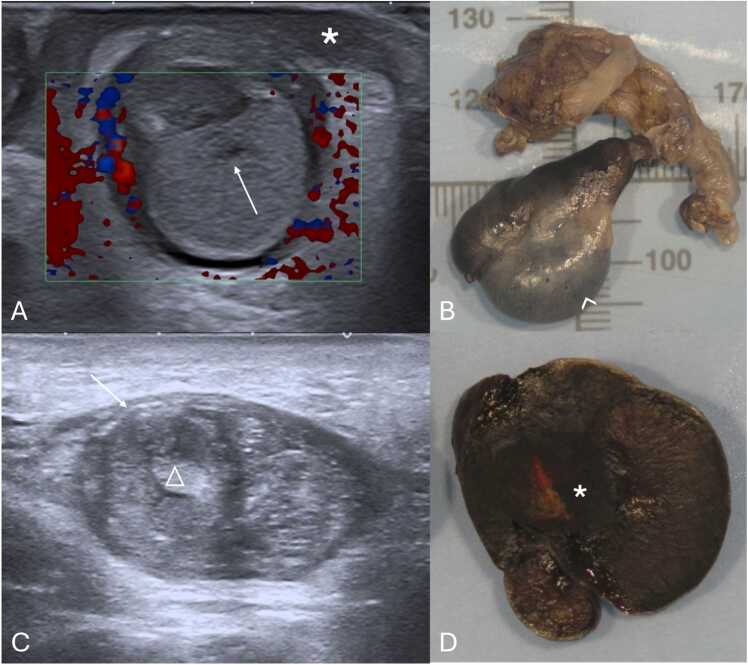
(A and B) Testicular acute extravaginal torsion in a 2-week-old neonate with left scrotal swelling. Testicular ultrasound in the transverse plane with color doppler image (A) depicting an enlarged testicle with slight heterogeneous echogenicity, hypoechoic striations (partially included in this image – arrow in A) and absent parenchymal vascular flow. Notice the presence of reactive thickening of the scrotal wall (* in A) as well as hyperemia of the adjacent tissues. Orchiectomy was performed, confirming the diagnosis of extravaginal torsion. The pathology specimen of the obtained testis is shown in (B), with abnormally bluish colored tissues, indicative of ischemia (arrowhead in B). (C and D) Testicular extravaginal chronic torsion in a 3-month-old neonate. Testicular ultrasound in the sagittal plane (C) at the ipsilateral inguinal canal, depicting an atrophied testicle with heterogeneous parenchymal echotexture, containing a hyperechoic calcification (△ in C) and calcification of the tunica albuginea (arrow in C). Pathology specimen photograph of the necrotized testis is shown in (D), with abnormal coloration and loss of typical parenchymal structure (* in D), indicative of necrosis.

Extravaginal torsion's sonographic features depend on the torsion stage (Table 3) [1], [58]. In acute stages, marked enlargement, heterogeneity, and vascularity loss are observed, alongside linear hypoechoic striations, subtunica fluid, and hydrocele (Fig. 10A and B) [1]. Later stages may display testicular atrophy with parenchymal heterogeneity (Fig. 10C and D) [1]. Calcification of the tunica albuginea is also common (Fig. 10C - arrow) [23]. These grayscale US changes are helpful in diagnosis because normal testicular flow may be undetectable in neonates [1], [59].

**Table 3:** Summarized imaging findings in extravaginal testicular torsion.

**Imaging Patterns of Extravaginal Testicular Torsion**	
Acute stages	− Marked testicular enlargement − Heterogeneity − Absent vascular flow
Later stages	− Testicular atrophy with parenchymal heterogeneity

#### 7.3.2 Intravaginal torsion

**Fig. 11:**
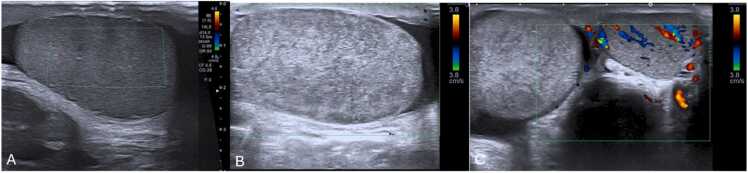
(A) Scrotal sagittal color-doppler US of a 12 year-old male with testicular torsion, obtained less than 6 h after symptom onset. Notice that although there is loss of vascularity, the testis has preserved echogenicity and homogenous echotexture, indicating the viability of the tissues. Compare with the sagittal and transverse images (B and C) of a testicular color-doppler US of another 17 year-old-male, obtained 12 h after symptom onset, showing an enlarged right testicle (volume of 24 cc vs. 13 cc of the left) with loss of vascular flow, and a heterogeneous parenchyma, containing areas of hyper- and-hypo echogenicity related to edema and swelling. Notice the difference in size, echotexture and vascularity of the right in comparison with the left testicle.

**Fig. 12:**
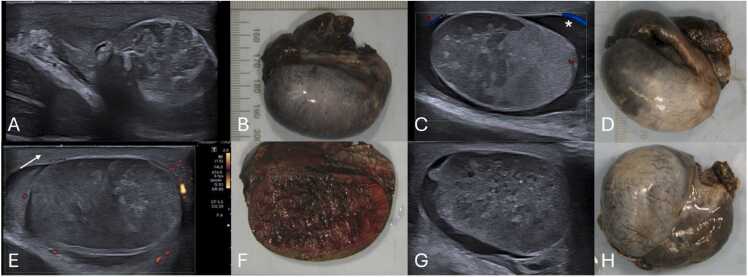
(A–H) Scrotal ultrasound and respective pathological specimens of different patients with complicated testicular torsions. Notice that the areas of testicular heterogeneity and loss of normal parenchymal echotexture represent necrotized testicular parenchyma. Note the presence of reactive hydrocele (* in C) as well as thickening of the scrotum wall (arrow in E), which are non-specific findings, sometimes seen.

Grayscale US findings are variable and evolve over time (Table 4) [22]. In the acute phase (first 4–6 h), the testicle may appear normal (Fig. 11A), becoming enlarged, hypoechoic, and heterogeneous due to edema and swelling over time (Fig. 11 B and C) [1], [22]. After 24 h without treatment, it becomes complicated [22]. Testicular heterogeneity favors nonviability, whereas a homogenous echotexture suggests viability (Fig. 12) [1], [60]. Other nonspecific findings sometimes may include thickening of the scrotum wall and reactive hydrocele (Fig. 12C and E).

**Table 4:** Summarized imaging patterns in intravaginal testicular torsion.

**Imaging Patterns of Intravaginal Testicular Torsion**	
Acute phase (first 4–6 h)	− Homogenous parenchymal echotexture, suggesting viability
During both phases	− Decreased or absent blood flow on Doppler-US
	− Whirlpool sign of a twisted spermatic cord (direct sign)
	− Enlargement of the epididymis with diminished blood flow
Later phases (after 6 h)	− Hypoechoic and heterogeneous echotexture, due to edema, indicating nonviability

**Fig. 13:**
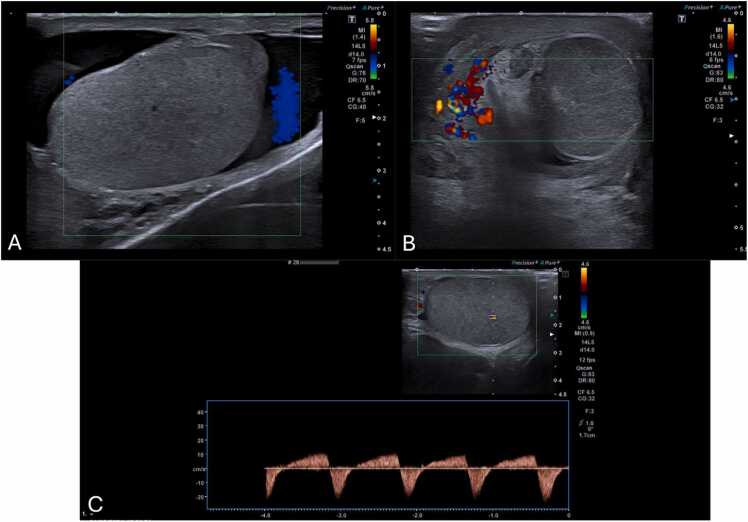
(A–C) Scrotal ultrasound in different male adolescent patients with surgically confirmed testicular torsions. (A and B) Sagittal and transverse color-Doppler US of torsed testes, with loss of normal blood flow on color-Doppler, depicting the typical ultrasonographic appearance of this condition. Notice that the parenchyma has as homogenous echotexture, indicating that the torsion is recent and the testis salvageable. (C) Sagittal testicular Spectral Doppler-US in a 16-year-old presenting with reduced testicular parenchymal color Doppler signal as well as abnormal high resistive blood flow (RI > 0,75), indicating the presence of obstruction to the normal blood flow.

**Fig. 14:**
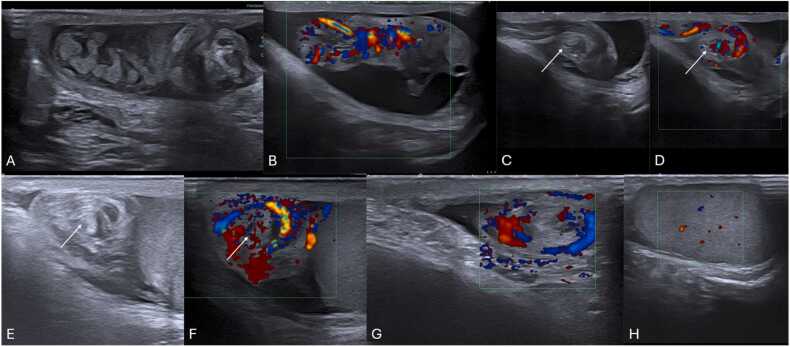
(A–H) Coupled testicular b-mode and color Doppler US images in the sagittal and transverse planes in different adolescent male patients with surgically proven testicular torsions. (A–F) Different examples of the twisted spermatic cord (“Whirlpool sign”), a direct sign of torsion. Note the twisting of the cord, particularly at the center of the twist (arrows). (G and H) Scrotal US images in a 15-year-old male, depicting the presence of a twisted spermatic cord (“Whirlpool sign”) with preserved testicular blood flow. The radiological diagnosis was suggested based on identification of the twisted spermatic cord. This case is an important reminder for the sonographer to systematically search for this sign in suspected testicular torsions.

**Fig. 15:**
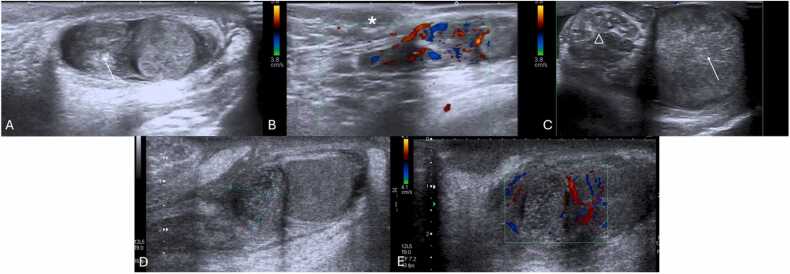
(A and B) Testicular ultrasound of a 6-year-old male with subacute scrotal pain. B-mode and color-Doppler ultrasound revealed an enlarged epididymis (arrow in A) with increased vascular flow on Color Doppler, allowing the diagnostic suspicion of epididymitis to be confirmed. Compare this with the scrotal color Doppler US transverse image of another 9-year-old male with acute, intense testicular pain. Notice the presence of enlarged epididymis (△ in C) and testicle (arrow in C) with absent flow, indicative of testicular torsion. (D and E) Testicular sagittal and transverse plane b-mode and color Doppler US images of a 12-year-old male with acute onset of testicular pain. On physical examination, a blue nodule adjacent to the painful area was present (no images available). US revealed a round, heterogeneous, avascular extra-testicular structure (between calipers in D), with preserved testicular vascular flow, allowing confirmation of the clinical suspicion of diagnosis of a torsed appendage.

Doppler-US is crucial for detecting changes in blood flow indicating torsion by showing decreased or absent flow (Fig. 13A and B) [22], [61]. However, false negatives can occur, in which an abnormally high resistive index (> 0.75) is depicted on Spectral Doppler (Fig. 13C), when there is intermittent or early torsion, as well as in boys 2–4 years of age, especially when the testis are in the inguinal canal [23], [62]. Thus, the most reliable and a direct sign of torsion of testicular torsion, the "whirlpool sign" (a twisted spermatic cord) (Fig. 14) [1], [22], [63], should always be sought along the entire route of spermatic cord, especially when vascularity appears normal (Fig. 14G and H) [1], [22]. It has been reported to have a sensitivity and specificity of 92 % and 99 %, respectively, in older children [23]. Additionally, enlargement of the adjacent epididymis with diminished or absent blood flow is extremely useful when present, distinguishing testicular torsion from acute epididymitis, in which blood flow is increased (Fig. 15A–C) [1], [22], [23].

## 8 Added value of CEUS in diagnosing gonadal torsion

**Fig. 16:**
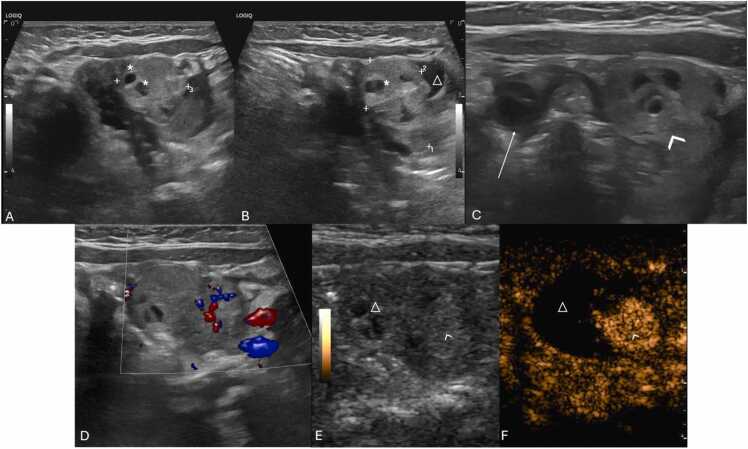
(A–F) Surgically confirmed ovarian torsion in a 4-month-old infant, with irritability and abdominal discomfort referred by the mother. Transabdominal pelvic ultrasound over the hypogastrium, revealed an asymmetrically enlarged, hyperechoic and inhomogeneous left ovary (between calipers in A and B) and containing several follicles (* in A and B). Notice the presence of free fluid around the left ovary (△ in B). Compare the difference in size and appearance between the right (arrow in C) and left ovaries (arrowhead in C). Doppler US of the left ovary (D) revealed absent vascular flow in the ovarian parenchyma, with preserved flow in what appeared to be a torsed vascular pedicle - raising suspicion for ovarian torsion. To confirm the diagnosis, CEUS was performed, showing no enhancement in the left ovary (△ in E and F) while maintaining normal enhancement of the associated vascular pedicle (arrowhead in E and F). Intraoperative findings confirmed an ischemic left ovary.

The advancement of diagnostic imaging techniques, particularly Contrast-Enhanced US (CEUS), has the potential to improve the accuracy and efficacy of diagnosing gonadal torsion [1], [7], [22], [46]. CEUS seems to help document absence of gonadal perfusion where traditional methods yield inconclusive results [22], [46], [64]. An observational study in 2021 involving 20 surgically confirmed cases of pediatric ovarian torsion found CEUS to have a 95 % accuracy rate, with a sensitivity of 94 % and specificity of 100 %, significantly outperforming the 81 % accuracy of conventional US [41], [65], [66]. By assessing the presence or absence of regular, symmetrical ovarian parenchymal enhancement following intravenous contrast administration, CEUS can effectively confirm or rule out torsion, respectively [66]. This added layer of diagnostic clarity becomes especially valuable in ambiguous cases where classical US signs are inconclusive, establishing CEUS as an invaluable decisive tool (Fig. 16). Furthermore, CEUS addresses several challenges that conventional US faces - such as the dual blood supply of the ovaries, partial or intermittent torsion, the small size and low flow velocity of pediatric vessels, and the gonads' deep anatomical locations - making it particularly beneficial in cases where operator experience is limited [1], [7], [67].

**Fig. 17:**
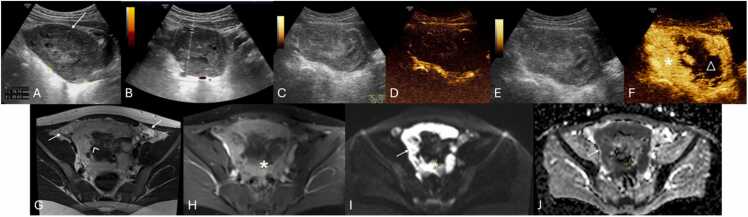
(A–J) Elective pelvic transabdominal ultrasound, CEUS, and pelvic MRI in a 9-year-old female presenting with pelvic pain for 15 days. She had previous a nephrectomy for Wilms tumor and was on follow-up. During ultrasound (A), a complex mass was noted in the pelvic floor (arrow in A), and the ovaries were not clearly visualized due to acoustic shadowing from adjacent bowels, raising concern for ovarian torsion with an associated adnexal mass. Due to MRI not being readily available, a CEUS was performed the same day (B–F), revealing a mass with intense peripheral enhancement (* in F) and no central enhancement (△ in F), likely due to necrotic components. The diagnosis of a pelvic tumor nature was placed. (G–J) Axial T2-weighted (G), contrast-enhanced T1-weighted FS and diffusion-weighted with respective ADC map (I and J) MR images obtained the following day are shown, confirming the presence of an independent pelvic mass (arrowhead in G) adjacent to the ovaries (arrow in G) with peripheral enhancement (* in H) and diffusion restriction (arrow in I and J). Surgery confirmed a 10 cm pelvic Wilms recurrence, with extensive necrosis, capsular rupture, and peritoneal implants.

Additionally, CEUS aids in evaluating gonadal masses by enabling identification of solid components (Fig. 17A–F), offering a practical alternative to contrast-enhanced MRI (Fig. 17G–J) [7], [46], [65], especially in emergency settings where access to MRI is limited [7], [46], [65]. Its advantages include technical simplicity, cost-effectiveness, speed, portability, and accessibility.

An argument for the role of CEUS in suspected testicular torsion can also be made, namely to detect perfusion in very young patients where intratesticular flow is physiologically reduced and difficult to detect on Doppler evaluation [65], [68].

**Fig. 18:**
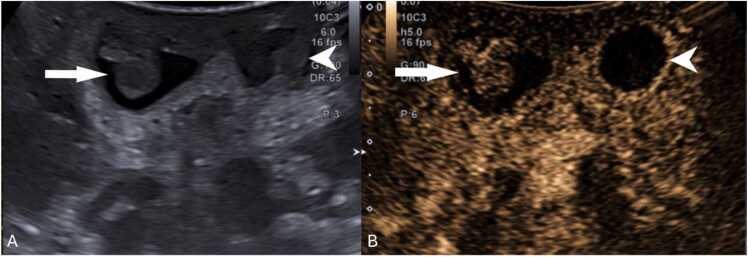
(A and B) Scrotal CEUS in a 3-day-old newborn with surgically confirmed testicular torsion. The left testicle appears reduced in volume compared to the right (arrowhead in A). CEUS reveals a complete absence of contrast enhancement in the left testicle, with increased vascularity in the surrounding peritesticular tissues (arrowhead in B), consistent with antenatal testicular torsion. Normal blood flow is visible in the right testicle (arrow in B).

Although Doppler US remains the primary tool for diagnosing acute testicular torsion due to its efficiency and reliability, CEUS might provide valuable information in specific scenarios. In cases of torsion up to 360º, where Doppler may still show some blood flow, CEUS aids diagnosis by revealing differential contrast uptake within the affected testicle [69]. Additionally, CEUS is particularly effective in detecting older torsions, where an atrophic testicle may still display intratesticular Doppler signals. By highlighting the absence of enhancement within the testicle and increased vascularity in the surrounding peritesticular tissues, CEUS enables a more confident diagnosis in these challenging cases (Fig. 18) [69]. Documentation of normal flow is especially relevant because it allows conservative management rather than immediate surgery to be proposed.

However, CEUS is not without challenges. The use of US contrast agents, although theoretically safe, is generally off-label for pediatric use in many regions, potently limiting its broader application [70]. Additionally, movement artifacts in younger patients - where obtaining steady images can be challenging - and partial torsion may result in false-negative or ambiguous results [65]. Despite these limitations, its ability to provide dynamic vascular imaging without ionizing radiation or iodinated contrast media makes it especially suitable for urgent settings, and underscores its value as a complementary or definitive alternative to conventional US [7], [46], [71], [72].

To provide a structured clinical decision-making framework, Table 5 was designed, highlighting each modality’s primary role, advantages, limitations, and optimal clinical scenarios, particularly in children. It underscores the essential roles of US, CEUS, and MRI in the diagnostic algorithm for gonadal torsion. While US is widely used as an initial imaging modality due to its availability and rapid results, CEUS provides enhanced diagnostic specificity in equivocal cases, particularly in assessing vascular perfusion. MRI, with its superior resolution and anatomical detail, remains invaluable for complex cases or when other modalities yield inconclusive results. Together, these tools form a complementary suite of diagnostic options, enabling tailored and effective management strategies for patients with suspected gonadal torsion.

**Table 5:** Summarized structured clinical decision-making table for pediatric gonadal torsion, highlighting the essential roles of US, CEUS, and MRI.

	**Conventional US**	**CEUS**	**MRI**
**Primary Role**	− First-line modality for evaluating vascularity and anatomy	− Real-time evaluation of vascular perfusion and gonadal viability	− Comprehensive anatomical characterization and evaluation of complex pathologies
**Key Advantages**	− Widely available and non-invasive − Rapid and cost-effective − No need for contrast agents	− Enhances diagnostic clarity in ambiguous cases − No ionizing radiation − Portable and accessible for bedside use	− Superior resolution for soft tissues − High diagnostic accuracy for complex cases − Less operator-dependent
**Limitations**	− Operator-dependent − May miss subtle perfusion changes − May provide inconclusive results in complex cases	− Off-label use, but considered safe in pediatric patients − Motion artifacts in younger children	− High cost and limited accessibility in an emergency setting − Longer acquisition times (may need sedation) − Contraindicated in patients with specific implants or severe claustrophobia
**Optimal Clinical Scenarios**	− Initial evaluation of suspected torsion − Differentiation of torsion from other causes of acute scrotal or pelvic pain	− Ambiguous findings on Doppler ultrasound − Suspected partial or intermittent torsion − Small gonads with low vascular flow − Acute settings where MRI is not readily accessible	− Inconclusive ultrasound or CEUS results − Characterization of adnexal or scrotal masses − Differential diagnosis of complex pelvic pathologies

In the pediatric population, the added value of CEUS in the evaluation of gonadal torsion remains to be fully substantiated, with ongoing debates about its significant added value over conventional methods. To further substantiate the role of CEUS in pediatric radiology, additional studies are necessary [68].

## 9 Value of US elastography in diagnosing gonadal torsion

In children under 10 years of age, testicular volume increases while testicular stiffness consistently decreases with weight gain. Normal stiffness values have been estimated at 3.9 kPa for children aged 15.8 months and 3.1 kPa for those aged 8 years [73].

Shear wave elastography (SWE) typical findings of testicular torsion include the "stiff ring sign" in the testis, the "stiff knot sign" in the spermatic cord, and increased stiffness of the testicular capsule and twisted spermatic segment [74]. Such findings, particularly increased stiffness, are clinically valuable for distinguishing testicular torsion from acute orchitis. Normal SWE values serve as a critical baseline in these differential diagnoses.

A paper that evaluated the potential of elastography for follow-up in testicular salvage after torsion, involving both children and adults, suggests that elastography could become a practical and feasible complementary tool for assessing testicular viability post-torsion [75].

Currently, no published studies explore the use of US elastography of the ovaries in pediatric or adult populations, nor its application in diagnosing ovarian torsion.

## 10 Management

### 10.1 Ovarian

In pediatric patients, the primary management goal is to preserve ovarian tissue [14]. Acute cases are treated with emergency laparoscopic surgery for detorsion and to assess ovary viability [14]. Conservative surgery is performed to maintain ovarian function, with salpingo-oophorectomy reserved for clear necrosis and structural loss [10], [14]. Conservative management, including detorsion, removal of cysts to prevent recurrence (beyond simple drainage), and preservation of the adnexa, has proven safe and effective [10], [33], [53]. Notably, even ovaries that appear necrotic at surgery can show signs of recovery, with some studies demonstrating normal follicular development after only six weeks [4], [10], [14].

For visible cysts post-detorsion, options include cystectomy, fenestration, or aspiration [14]. Masses that raise concerns for malignancy warrant salpingo-oophorectomy [14]. Oophoropexy post-detorsion is debated due to the lack of long-term efficacy and safety data [10], [14], [33].

Management in fetal and neonatal populations is more controversial, often linked to underlying cysts [14], [76]. While cysts under 5 cm may spontaneously regress, larger cysts carry a higher torsion risk [14], [33], [54], [77]. Some suggest in utero aspiration for large simple cysts to prevent torsion, though this approach is rarely used due to various concerns, including procedural risks [14], [76]. Conversely, conservative observation and surgical intervention for complex or symptom-causing cysts that do not spontaneously resolve are advocated by many, highlighting a cautious approach to these vulnerable populations [14], [19].

### 10.2 Testicles

Intravaginal testicular torsion prompts immediate surgical exploration upon suspicion or diagnosis [1], [2], [5], [7]. During surgery, the affected testes are carefully examined; if torsion is confirmed, an attempt is made to detorse and evaluate the testes for viability [2]. Viable testes are then subjected to orchidopexy, a preventive measure against the substantial risk—estimated at 40 %—of metachronous torsion occurring in the contralateral testis [2], [78]. Conversely, testes found to be necrotic or showing no signs of recovery post-detorsion are typically removed through orchiectomy [2].

Treatment for prenatal testicular torsion is widely debated due to several complicating factors [5], [79]. Primary considerations include the timing and necessity of surgical exploration and how to approach the contralateral testicle, with decisions influenced by the child’s age at diagnosis and their overall health condition [5]. The reluctance to intervene surgically lies in the minimal likelihood of salvaging the torsed testicle, increased risks associated with neonatal anesthesia, and the potential for inadvertently damaging the contralateral testicle during surgery [5], [80]. The age at diagnosis is particularly important because the risk of asynchronous torsion decreases significantly after the tunica vaginalis adheres to the dartos muscle, typically around 4–6 weeks of life, reducing the necessity for contralateral orchidopexy, as it theoretically eliminates the risk of future torsion on the unaffected side [5], [73], [81].

## 11 Clinical outcomes

### 11.1 Ovarian

Conservative laparoscopic management for ovarian torsion preserves ovarian function in pediatric patients, ensuring normal progression through puberty and maintaining future fertility potential [4], [10], [34]. For long-term monitoring, surveillance ultrasounds are recommended at 3-month intervals post-surgery for adnexal torsion and/or cystectomy, followed by semi-annual to annual checks to assess for cyst recurrence and ovarian health [10].

In menarchal patients with a history of functional ovarian cysts, hormonal therapy may be employed to decrease ovulation frequency and the risk of cyst recurrence, thereby diminishing the likelihood of further adnexal torsion [10]. However, hormonal therapy does not impact the recurrence of paraovarian/paratubal cysts or dermoid ovarian cysts, as these are not linked to the ovulation process [10].

### 11.2 Testicular

The sooner a testicular torsion is corrected, the better the chances of saving the testicle, with a 90 % salvage rate before 12 h from onset [5], [6], [7], [23]. This rate decreases to 54 % if treatment occurs between 13 and 24 h and drops further to 18 % after 24 h [1], [6], [23], [46]. It is therefore critical to avoid delays in treatment, particularly those resulting from repeat imaging tests, as these increase the likelihood of orchiectomy [7], [20], [46], [75].

## 12 Differential diagnoses

### 12.1 Ovarian

**Fig. 19:**
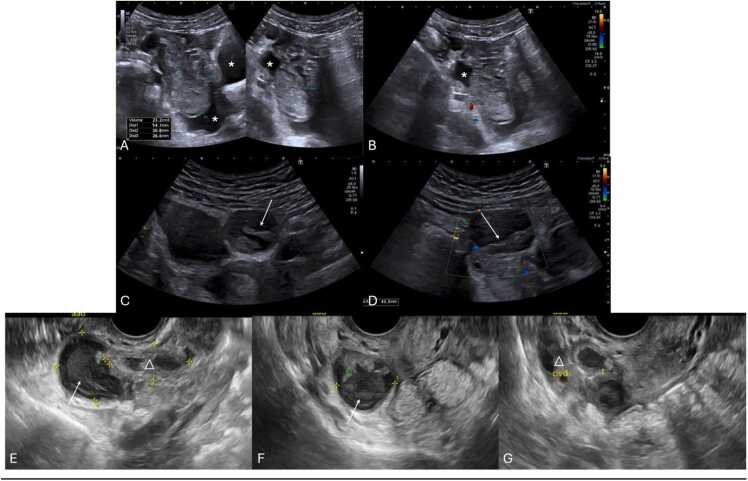
(A and B) Pelvic transabdominal b-mode and color Doppler US images in a 12-year-old female with acute onset of pelvic pain, suspected of ovarian torsion. US revealed a large, voluminous (23 cc) complex adnexal mass (between calipers in A), with no vascular flow, with associated free fluid in de the pelvic cavity (* in A and B). Due to clinical and radiological findings consistent with ovarian torsion, she was submitted to exploratory laparoscopy, which revealed the presence of a ruptured hemorrhagic ovarian cyst with no signs of ovarian torsion. (C and D) Pelvic transabdominal sagittal and transverse b-mode and color Doppler US in a 14-year-old female with acute onset of pelvic pain. US reveals a complex cystic mass with no vascular flow in the ovary, as well as the presence of the retractable cloth sign (arrow in C and D), characteristic of a hemorrhagic cyst. (E–G) Transvaginal pelvic ultrasound in an adolescent female with pelvic pain and associated fever and leukocytosis. US revealed a complex mass (arrow in E and F) adjacent to the right ovary (triangle in E and G) as well as dilated fallopian tube (t in G), allowing the diagnosis of a tubo-ovarian abscess to be made.

Ovarian torsion's imaging findings can be mimicked by several conditions, complicating accurate diagnosis [14]. In adolescents, hemorrhagic cysts are common mimics, presenting with echogenic appearances on US that resemble torsed ovaries, especially when patients report severe abdominal pain (Fig. 19A–D) [14], [82]. Similarly, tubo-ovarian abscesses and ovarian neoplasms can cause pain and manifest as heterogeneous adnexal masses on US, further complicating the distinction from torsion (Fig. 19 E–G).

The challenge increases in neonates, where the highly variable US appearance of ovarian torsion makes it difficult to differentiate from other complex cystic masses commonly seen in this demographic [14]. Conditions such as hematocolpos, duplication cysts, urachal remnants, meconium pseudocysts, and lymphatic malformations may display US features akin to those of neonatal ovarian torsion, requiring careful evaluation to ensure accurate diagnosis.

### 12.2 Testicular

Differentiating testicular torsion from conditions that present with acute scrotal pain such as epididymitis and appendage torsion is critical. Consideration of clinical symptoms and imaging features is essential for an accurate diagnosis.

Epididymitis typically presents on grayscale US with a swelled epididymis that displays variable or heterogeneous echogenicity, with increased flow within the epididymis, testis, or both on color Doppler US (Fig. 15 A and B) [1], [22], [23]. Analysis of the epididymal waveform often reveals a low-resistance pattern compared to the absent or high-resistance pattern seen in testicular torsion [1], [23].

Testicular appendages are prone to torsion due to their sessile nature [23]. On physical examination a blue nodule can be seen on the skin adjacent to the painful area, known as the “blue dot sign”, which is specific [1], [83]. On imaging, a torsed appendage appears as a round, avascular extra-testicular structure with variable echogenicity (Fig. 15 D and E) [1], [23]. Unlike testicular torsion, testicular vascularity remains unaffected in appendage torsion.

## 13 Conclusion

Gonadal torsion in the pediatric population presents a significant clinical challenge, requiring prompt diagnosis and intervention to preserve future reproductive capabilities. This comprehensive review underscores the critical nature of early diagnosis and the strategic use of imaging techniques, such as US and CEUS to enhance diagnostic accuracy.

Key takeaways include the necessity for a high index of suspicion, particularly given the nonspecific nature of clinical presentations in regard to ovarian torsion. The management strategies for ovarian and testicular torsion emphasize the importance of conservative surgical intervention where possible, aiming to salvage the gonad while minimizing the risk of future recurring events. We also highlight the value this review provides for radiologists, especially trainees, serving as an easily accessible educational resource.

The promising potential of CEUS as a diagnostic tool, particularly as a non-invasive, cost-effective, and accessible alternative to MRI in acute settings, is also highlighted. Future research should focus on comparing CEUS with MRI to validate its diagnostic accuracy and effectiveness, especially in the ovarian torsion, thus potentially transforming the diagnostic landscape and improving patient outcomes.

The incorporation of CEUS into the diagnostic workflow offers a promising option for diagnosing gonadal torsion. This, coupled with an understanding of the condition's epidemiology, pathophysiology, and clinical presentation, can significantly impact the outcomes for pediatric patients.

## CRediT authorship contribution statement

**Silva Ana Catarina:** Resources. **Sá Pedro:** Writing – review & editing, Investigation, Formal analysis. **Soares-Aquino Carolina:** Resources, Methodology, Formal analysis, Conceptualization. **Ključevšek Damjana:** Formal analysis, Investigation, Resources, Validation, Writing – review & editing. **Costa Dias Sílvia:** Writing – review & editing, Supervision, Resources, Project administration, Methodology, Data curation, Conceptualization. **Freitas Inácio:** Writing – review & editing, Writing – original draft, Resources, Project administration, Methodology, Investigation, Formal analysis, Data curation, Conceptualization.

## Statements

• No funds, grants, or other support was received.
• All authors certify that they have no affiliations with or involvement in any organization or entity with any financial interest or non-financial interest in the subject matter or materials discussed in this manuscript.

## Declaration of Competing Interest

The authors declare that they have no known competing financial interests or personal relationships that could have appeared to influence the work reported in this paper.
