# Supplementary material for: Pediatric gonadal torsion in radiology: A comprehensive literature and pictorial review using surgically proven cases
Source: Eur J Radiol Open. 2025 Mar 20;14:100644. doi: 10.1016/j.ejro.2025.100644 (PMC11978341; doi:10.1016/j.ejro.2025.100644)
Supplement: Supplementary file 1 — Supplementary material [file mmc1.docx]

| **Patient-image correspondance** | **Age at surgical procedure** | **Relevant medical history** | **Symptoms** | **Duration of symptoms** | **Referral** | **First imaging method** | **Second imaging method** | **Radiological diagnosis** | **Surgical procedure** | **Result** |
| --- | --- | --- | --- | --- | --- | --- | --- | --- | --- | --- |
| Figure 2 | 12 y | - | Intermittent abdominal pain, nausea and vomiting | 6 m | Clinic | MRI | - | Left ovary torsion | Laparoscopic detorsion of left ovary | Atrophy of left ovary |
| Figure 4 | 13 y | First episode of ovarian torsion one year before | Abdominal pain with left side tenderness | 3 d | Emergency | US | CT | Left ovary torsion | Laparoscopic detorsion of left ovary and oophoropexy | Relapse one year after, new laparoscopic detorsion |
| Figure 5 (A-B) | 13 y | - | Abdominal pain and vomiting | 12 h | Emergency | US | - | Right ovary torsion | Laparoscopic detorsion of right ovary and drainage of simple cyst | Right ovary teratoma detected 3 years after, excised with ovarian preservation |
| Figure 5 (C) | 14 y | - | Abdominal pain and vomiting | 8 h | Emergency | US | - | Left ovary torsion with complex solid mass | Laparoscopic detorsion of left ovary and excision of ovarian teratoma with ovarian preservation | Discharged from clinic after 2 years, no atrophy |
| Figure 5 (D-E) | 12 y | - | Abdominal pain and vomiting | 3 d | Emergency | US | CT | Left ovary torsion | Laparoscopic detorsion of left ovary, marsupialization of cyst and biopsy | Lost for follow-up |
| Figure 5 (F) | 14 y | - | Left lower abdominal pain, biliary vomiting | 18 h | Emergency | US | CT | Left ovary torsion | Laparoscopic detorsion of left adnexa, tubal cyst drainage | Under follow-up, no atrophy |
| Figure 5 (G-H), Figure 6 (B-G), Figure 7 (A-D) | 8 y | - | Left lower abdominal pain, anorexia, vomiting | 5 m with worsening in the last month | Emergency | US | MRI | Bilateral ovarian torsion | Laparoscopic left salpingofoorectomy due to necrosis of left adnexa; normal right ovary | Second episode, on the right ovary |
| Figure 5 (G-H), Figure 6 (B-G), Figure 7 (A-D) | 11 y | Previous left (at 8 years) and right (at 10 years) ovarian torsion; previous right oophoropexy | Right lower abdominal pain, vomiting and nausea | 5 d, worsening on the last day | Emergency | US | MRI | Right ovary torsion | Laparoscopic detorsion of right ovary torsion, no sign of the first oophoropexy material | Relapse on the right side, oophoropexy 1 year after in a torsion episode |
| Figure 6 (A) | 14 y | - | Intermittent right abdominal pain. Asymptomatic upon examination | 7 m | Clinic | US | - | Right ovary torsion | Laparoscopic detorsion of right ovary, cystectomy | Discharged from clinic after 5 years, smaller right ovary |
| Figure 7 (E-F) | 13 y | Cerebral palsy and West syndrome | Right lower abdominal pain | 3 d | Emergency | US | - | Right ovary torsion | Laparoscopic detorsion of right ovary. Relapse on the next few hours, submitted to laparoscopic oophorectomy due to necrosis | Discharge from clinic after 2 months |
| Figure 8 (A) | 8 d | On day 3, ultrasound suspicious of enteric duplication | Slight feeding intolerance, no abdominal pain | 8 d | Neonatology ward | US | - | Enteric duplication cyst | Laparoscopic right oophorectomy | Discharge from clinic after 4 years |
| Figure 8 (B-D) | 2 m | Prenatal diagnosis of bilateral ovarian cysts (5cm on the left and 4cm on the right) | Feeding intolerance, vomiting, no abdominal pain | 10 d | Emergency | US | - | Left ovary torsion | Open detorsion left ovary and cystectomy through Pfannenstiel incision | Left ovary atrophy; discharged from clinic 2 years after. Involution of the right ovarian cyst. |
| Figure 9 (A-D) | 6 d | Prenatal diagnosis of left ovarian cyst of enteric duplication | Biliary vomiting, no abdominal pain | 4 d | Neonatology ward | US | - | Bilateral ovarian cysts, suspicion of left ovary torsion | Laparoscopic left oophorectomy and puncture of right ovary cysts | Discharged from clinic after 2 years; involution of right ovary cysts |
| Figure 16 | 4 m | - | Abdominal pain and vomiting feeding refusal | 8 d, worsening the last day | Emergency | US | US with CEUS | Left ovarian torsion | Laparoscopic detorsion of left ovary and tube | Asymptomatic, under follow-up |
| Figure 17 | 9 y | Wilms tumor, previous right nephroureterectomy and chemotherapy | Lower abdominal pain | 10 d | Emergency | US and CEUS | CT (emergency), followed by elective MRI | Pelvic relapse of Wilms tumor | Elective resection of pelvic Wilms tumor | Under follow-up |
| Figure 19 (A-B) | 12 y | Asthma | Lower abdominal pain | 3 h | Emergency | US | - | Right ovary torsion | Laparoscopic exploration, identification of rupture of hemorrhagic cyst of the right ovary without torsion | Lost for follow-up |
| Figure 19 (C-D) | 14 y | - | Lower abdominal pain. Vomiting, | 1 w | Emergency | US | - | Hemorrhagic cyst of the left ovary | Not submitted to surgery | Lost for follow-up |
| Figure 19 (E-G) | 16 y | Esophageal atresia; appendectomy | Pelvic pain; vaginal discharge | 5 d | Emergency | US | Transvaginal US | Tubo-ovarian abscess (Inflammatory pelvic disease) | Not submitted to surgery | Under gynecology follow-up (Pyosalpinx by *Chlamydia trachomatis*) |

Supplementary Table 1- Demographic, Clinical, and Imaging Data for Surgically Proven Gonadal Torsion Cases. This table provides an overview of all pediatric cases with surgically confirmed gonadal torsion at our institution between 2011 and 2024. It includes each patient’s age at the torsion episode, sex, presenting symptoms, imaging modalities performed (and their findings), surgical procedures, final operative diagnoses, outcomes, and follow-up details.
